# Supplementary material for: Tuning of lattice oxygen reactivity and scaling relation to construct better oxygen evolution electrocatalyst
Source: Nat Commun. 2021 Jun 28;12:3992. doi: 10.1038/s41467-021-24182-w (PMC8238955; doi:10.1038/s41467-021-24182-w)
Supplement: Supplementary file 1 — Supplementary information [file 41467_2021_24182_MOESM1_ESM.pdf]

SUPPLEMENTARY INFORMATION

Supplementary Methods

Materials. $NaNO_3$ (99%), $MnCO_3$ (99%), Nafion solution (~5 wt.%), tetramethylammonium hydroxide (TMAOH, ~25 wt.% in H_2O) and D_2O (≥99.96 at.% D) were obtained from Sigma-Aldrich. Potassium iodide (≥99%), sodium thiosulfate (≥99%), potassium iodate (≥99%) and hydrochloric acid (37%) were obtained from Fisher Scientific. Absolute ethanol and isopropanol were obtained from the Chemicals Testing and Calibration Laboratory. All chemicals were used as received. High-purity water (18.25 MΩ·cm) supplied by a Milli-Q Gradient A10 system was used in all of the experiments.

Free energy calculations. To better reveal the factors that influence the catalytic trends of different materials, the linear scaling relations (LSRs) of adsorption energies between different intermediates centred on metal ions (*) are introduced[1]. In this way, a descriptor derived from the adsorption energy such as ΔG_{*O} - ΔG_{*OH} or the other physical or chemical property are facile to calculate and reflect the energy landscape of a reaction system[1,2]. The Gibbs free energy changes for the water oxidation steps were calculated using the following Equations (S1-S4), respectively[2,3],

$$\Delta G_1 = \Delta G_{*OH} - eU + \Delta G_{pH} \quad (S1)$$

$$\Delta G_2 = \Delta G_{*O} - \Delta G_{*OH} - eU + \Delta G_{pH} \quad (S2)$$

$$\Delta G_3 = \Delta G_{*OOH} - \Delta G_{*O} - eU + \Delta G_{pH} \quad (S3)$$

$$\Delta G_4 = 4.92\ eV - \Delta G_{*OOH} - eU + \Delta G_{pH} \quad (S4)$$

where U is the potential measured against normal hydrogen electrode (NHE) at standard condition (T = 298.15 K, P = 1 bar, pH = 0)[4]. The free energy change of the protons relative to the above-specified electrode at non-zero pH is represented by Nernst equation as $\Delta G_{pH} = -k_BT \ln(10) \times pH$. The Gibbs free energy differences of these intermediates include zero-point energy (ZPE) and entropy corrections (listed in Supplementary Table 10) according to $\Delta G_i = \Delta E_i + \Delta ZPE_i - T\Delta S_i$, where the energy differences ΔE_i are calculated with respect to H_2O and H_2 (at U = 0 and pH = 0). The theoretical overpotential is defined as the lowest potential at which all reaction steps are thermodynamically downhill[2,5].

Supplementary Tables

Supplementary Table 1 | Computed magnetization moment and charge of oxygen ions in $Na_xMn_3O_7$. Magnetization moment results from the presence of unpaired electrons in materials, and the fully paired electrons lead to a net magnetic moment of zero. For $Na_2Mn_3O_7$, the oxygen ions tend to form 8-electron stable structures with no unpaired electrons. When the number of Na^+ decreases from x=2, lattice oxygen ions are activated and oxidized, meanwhile Mn would be in their maximum achievable oxidation states of 4+. Thus, the unpaired electrons are generated in such oxidized oxygen ions.

Model	Magnetization moment (μB)	Charge (e)
$Na_2Mn_3O_7$-S1	0.11	-1.03
$Na_{1.5}Mn_3O_7$-S2	0.17	-1.00
$Na_{1.5}Mn_3O_7$-S3	0.23	-0.94
$NaMn_3O_7$-S4	0.30	-0.89
$Na_{0.5}Mn_3O_7$-S5	0.34	-0.80
$Na_{0.5}Mn_3O_7$-S6	0.60	-0.66

Supplementary Table 2 | Data for free energy change in various models for $Na_xMn_3O_7$.

Model	ΔG_1 (eV)	ΔG_2 (eV)	ΔG_3 (eV)	ΔG_4 (eV)	P1 (eV)	P2 (eV)	η (eV)
$Na_2Mn_3O_7$-S1	0.61	0.67	2.17	1.47	0	-0.40	0.94
$Na_{1.5}Mn_3O_7$-S2	0.51	1.24	1.60	1.57	0.57	-0.40	0.37
$Na_{1.5}Mn_3O_7$-S3	0.34	1.44	1.68	1.47	0.77	-0.14	0.45
$NaMn_3O_7$-S4	0.46	1.56	1.54	1.36	0.89	-0.14	0.33
$Na_{0.5}Mn_3O_7$-S5	0.71	1.64	1.48	1.08	0.97	-0.14	0.41
$Na_{0.5}Mn_3O_7$-S6	0.87	1.81	1.43	0.82	1.14	0	0.58
Ideal	1.23	1.23	1.23	1.23	0.56	-0.78	0

Supplementary Table 3 | Data for free energy change in various assumed models based on the scenario without considering the Na^+-mediated geometric effect (that is, P2 is fixed to 0, whereas P1 is varied due to the Na^+-mediated electronic effect.).

Assumed model	ΔG_1 (eV)	ΔG_2 (eV)	ΔG_3 (eV)	ΔG_4 (eV)	P1 (eV)	P2 (eV)	η (eV)	η_{min} (eV)

S1	0.61	0.67	2.57	1.07	0	0	1.34	0.39
S2	0.51	1.24	2.00	1.17	0.57	0	0.77	0.39
S3	0.34	1.44	1.80	1.35	0.77	0	0.57	0.39
S4	0.46	1.56	1.68	1.22	0.89	0	0.45	0.39
S5	0.71	1.64	1.60	0.96	0.97	0	0.41	0.39
S6	0.87	1.81	1.43	0.82	1.14	0	0.58	0.39

Supplementary Table 4 | Data for free energy change in various assumed models based on the scenario without considering the Na^{+}-mediated electronic effect (that is, P1 is fixed to 0, whereas P2 is varied due to the Na^{+}-mediated geometric effect.).

Assumed model	ΔG_1 (eV)	ΔG_2 (eV)	ΔG_3 (eV)	ΔG_4 (eV)	P1 (eV)	P2 (eV)	η (eV)	η_{min} (eV)
S1	0.61	0.67	2.17	1.47	0	-0.40	0.94	0.19
S2	-0.06	0.67	2.17	2.14	0	-0.40	0.94	0.19
S3	-0.43	0.67	2.45	2.24	0	-0.14	1.22	0.33
S4	-0.43	0.67	2.43	2.25	0	-0.14	1.20	0.33
S5	-0.26	0.67	2.45	2.05	0	-0.14	1.22	0.33
S6	-0.27	0.67	2.57	1.96	0	0	1.34	0.39

Supplementary Table 5 | Data for free energy of ΔG_{*OH}, ΔG_{*O}, ΔG_{*OOH} in various models for $Na_xMn_3O_7$.

Model	ΔG_{*OH} (eV)	ΔG_{*O} (eV)	ΔG_{*OOH} (eV)
S1	0.61	1.28	3.45
S2	0.51	1.75	3.35
S3	0.34	1.78	3.46
S4	0.46	2.02	3.56
S5	0.71	2.35	3.83
S6	0.87	2.68	4.11

Supplementary Table 6 | Na/Mn mole ratio (y, measured from ^{a}ICP-OES and ^{b}ICP-MS) and BET surface area (SA) of $Na_xMn_3O_{7-\delta}$.

x in $Na_xMn_3O_{7-\delta}$	$^a y$	$^b y$	SA (m^2g^{-1})
2.0	0.66	0.66	1.10
1.5	0.50	0.51	1.42
1.0	0.34	0.33	1.55
0.70	0.23	0.24	1.98

Supplementary Table 7 | Fitting results of Mn K-edge EXAFS curves. ($^a R$: bond distance; $^b CN$: coordination numbers; $^c\sigma^2$: Debye-Waller factors; $^d S_0^2$: Amplitude attenuation factors.)

Sample	bond	$^a R$ (Å)	$^b CN$	$^c\sigma^2$(Å^2)	$^d R$ factor
$Na_2Mn_3O_7$	Mn-O	1.907	6.0 (fixed)	0.005	0.016
$Na_{1.5}Mn_3O_7$	Mn-O	1.910	5.78	0.005	0.012
$NaMn_3O_7$	Mn-O	1.914	5.31	0.005	0.023
$Na_{0.7}Mn_3O_7$	Mn-O	1.918	4.86	0.005	0.032

Supplementary Table 8 | Summary of the results from ICP-MS measurements of the electrolyte collected from stability tests on $Na_xMn_3O_7$. The reported percentages refer to the mass of the dissolved element with respect to the initial element mass in the electrode.

	Na	Mn
$Na_2Mn_3O_7$	5.10%	0.11%
$Na_{1.5}Mn_3O_7$	0.23%	0.10%
$NaMn_3O_7$	0.17%	0.13%
$Na_{0.7}Mn_3O_7$	0.10%	0.11%

Supplementary Table 9 | Comparison of free energy change for deprotonation of *O and formation of *OOH on 1×1, 1×2 and 2×2 $NaMn_3O_7$ supercell. The overpotential of potential-determining step is also shown.

Model	ΔG_2 (eV)	ΔG_3 (eV)	η (eV)
1×1	1.556	1.544	0.326
1×2	1.555	1.543	0.325
2×2	1.24	1.60	0.37

Supplementary Table 10 | The calculated zero-point energy and entropy correction values (in eV).

	E	ZPE	TS	G
H_2	-6.99	0.28	0.40	-7.12
H_2O	-14.15	0.57	0.67	-14.25

Supplementary Figures

$Na_{2x/7}[Mn_{6/7}\square_{1/7}]O_2$	x = 0	x = 1/7	x = 2/7	x = 3/7 →
h^O (mathematical)	0	1/28	1/14	3/28
h^O (spin density)	0.06	0.09-0.12	0.15	0.17-0.30
h^O (bader charge)	0.49	0.50-0.53	0.56	0.60-0.67

Supplementary Fig. 1 | The calculated number of oxygen hole per oxygen (h^O) based on crystal field theory, magnetization moment and bader charge analysis. In general formulation $A[M'_xM_{1-x}]O_2$, the average number of $|O_{2p}$ per oxygen, n, is directly linked to the x stoichiometry (that is, for x =0, 1/3 and 2/3, the average number of M around each O is three, two and one, respectively, thus leaving n = 0, 1 and 2 $|O_{2p}$ per oxygen in each structure.). Note the calculated h^O is different from but the trend is in accordance with the mathematical prediction from crystal field theory, because all transition metals make iono-covalent interactions with oxygen in DFT calculations.

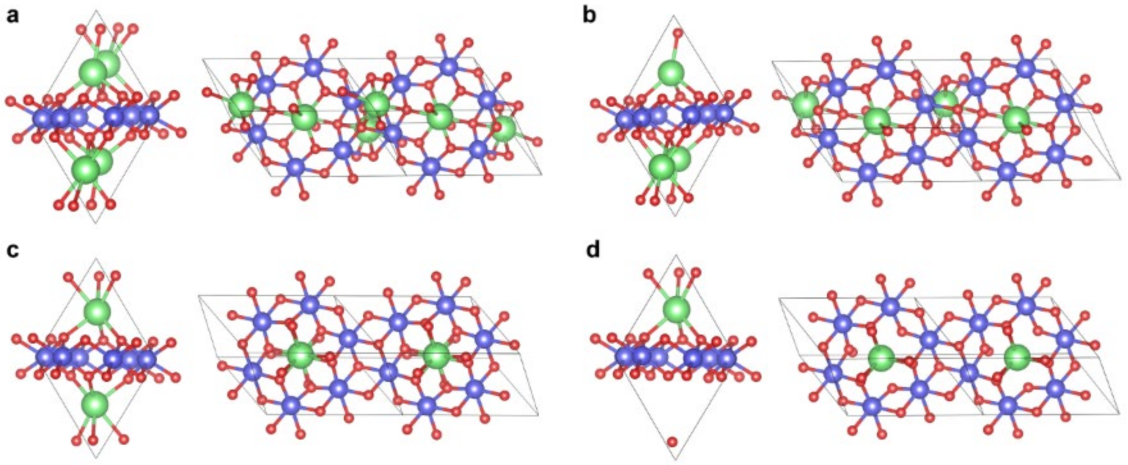

Supplementary Fig. 2 | Bulk models of $Na_xMn_3O_7$ with side view (left) and top view (right). (**a,** x = 2, **b,** x = 1.5, **c,** x = 1, **d,** x = 0.5; Blue, red and green spheres represent Mn, O and Na atoms, respectively.)

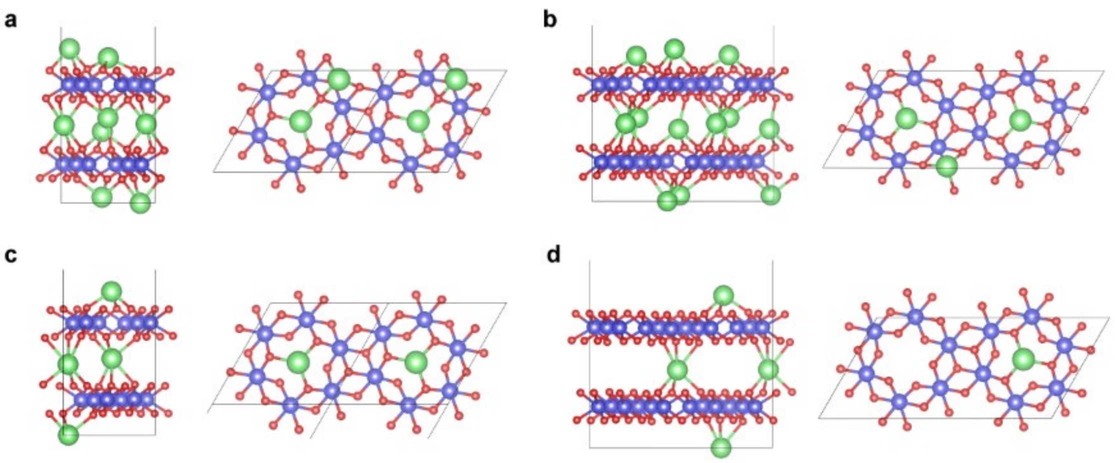

Supplementary Fig. 3 | Slab models of $Na_xMn_3O_7$ with side view (left) and top view (right). (**a,** $x = 2$, **b,** $x = 1.5$, **c,** $x = 1$, **d,** $x = 0.5$; Blue, red and green spheres represent Mn, O and Na atoms, respectively.)

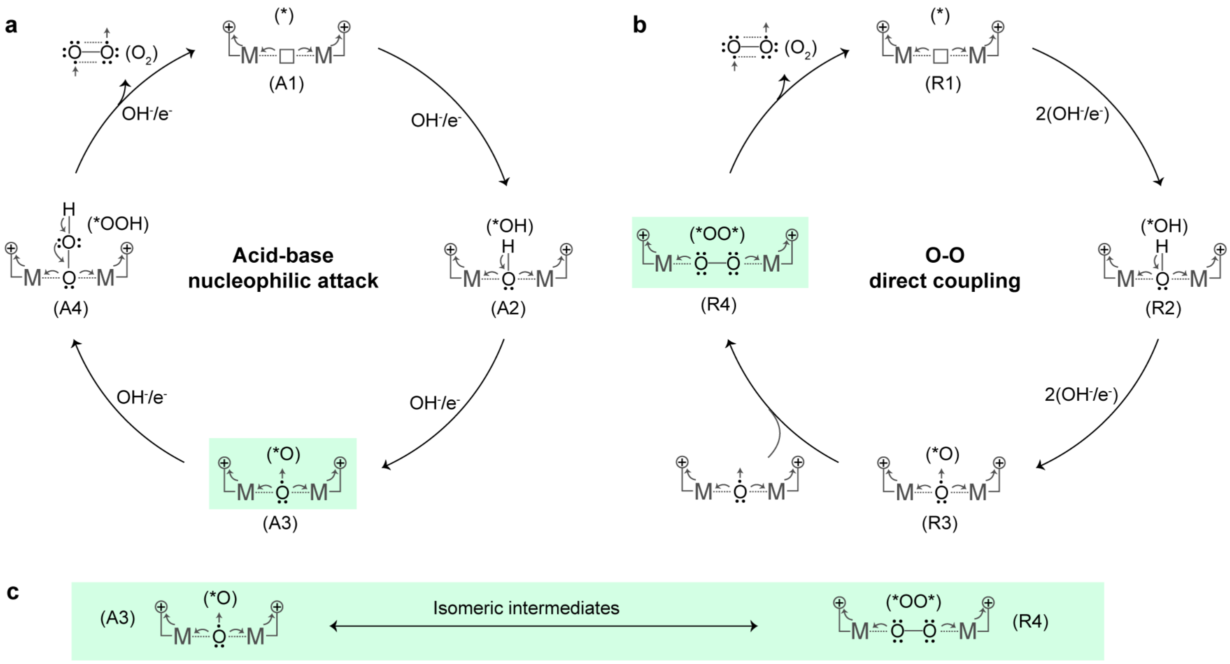

Supplementary Fig. 4 | Scheme of OER pathways including acid-base nucleophilic attack and O-O direct coupling, where A3 (*O) and R4 (*OO*) are the corresponding isomeric intermediates.

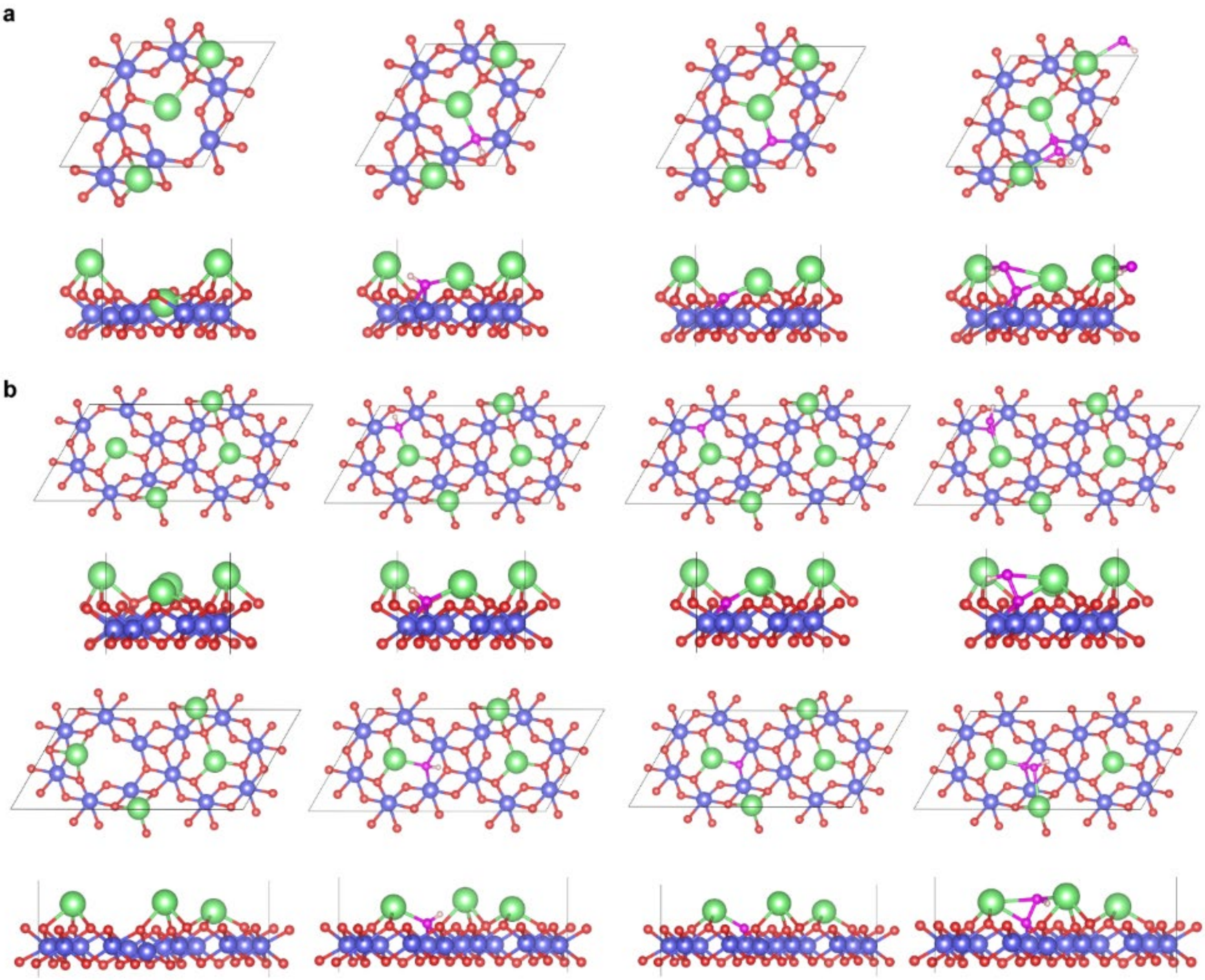

Supplementary Fig. 5 | Adsorption models of $Na_2Mn_3O_7$ (a) and $Na_{1.5}Mn_3O_7$ (b) for water oxidation. Blue, red and green spheres represent Mn, O and Na atoms, respectively. Pink spheres represent the oxygen atoms which are related to the intermediates of *OH, *O and *OOH.

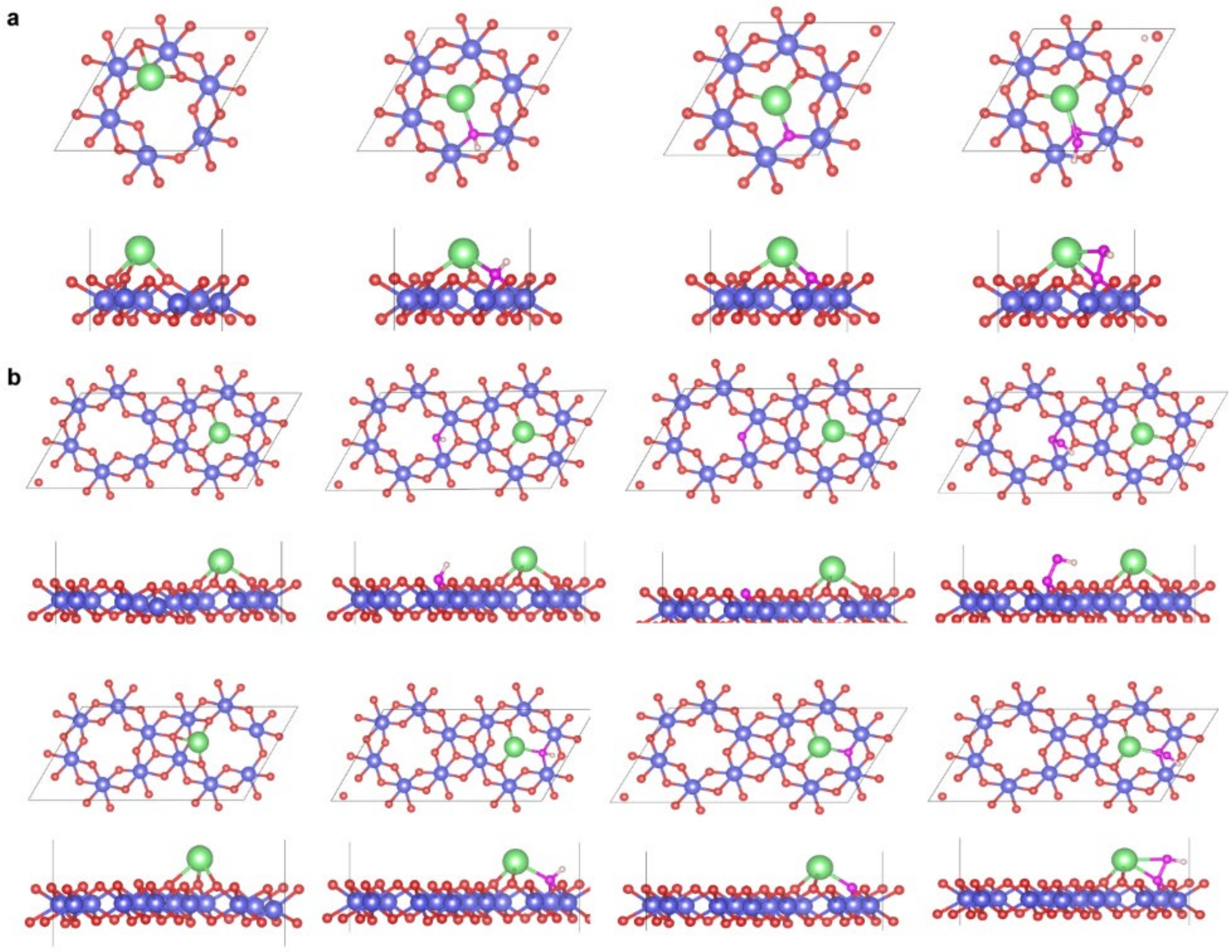

Supplementary Fig. 6 | Adsorption models of $NaMn_3O_7$ (**a**) and $Na_{0.5}Mn_3O_7$ (**b**) for water oxidation. Blue, red and green spheres represent Mn, O and Na atoms, respectively. Pink spheres represent the oxygen atoms which are related to the intermediates of *OH, *O and *OOH.

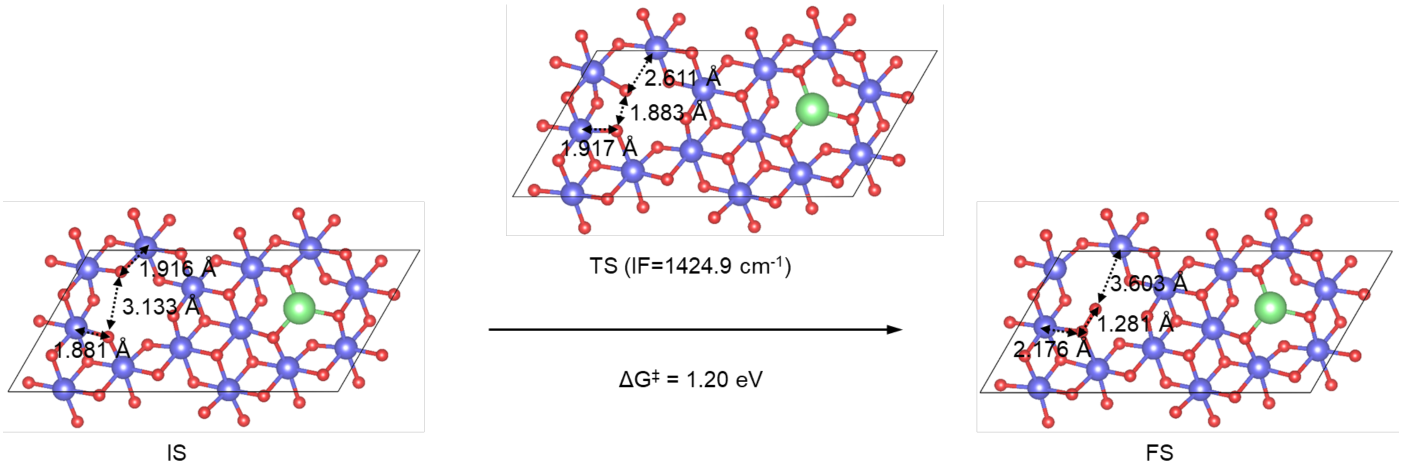

Supplementary Fig. 7 | Activation free energy ($\Delta G^{\ddagger}$) for O-O bond formation and corresponding adsorption and transition state structures on S_6 in $Na_{0.5}Mn_3O_7$ slab. (IS: initial state; TS: transition state; FS: final state; IF: imaginary frequency).

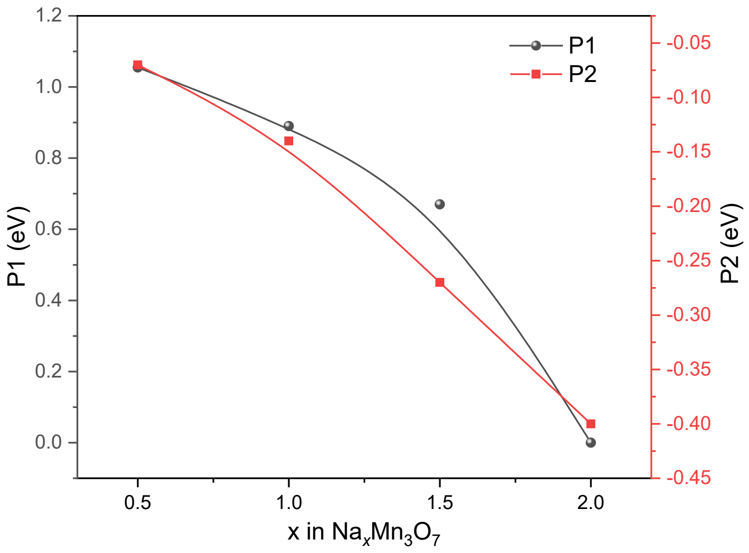

Supplementary Fig. 8 | Correlation between the parameters of P1 and P2 and the number of Na^+ in $Na_xMn_3O_7$.

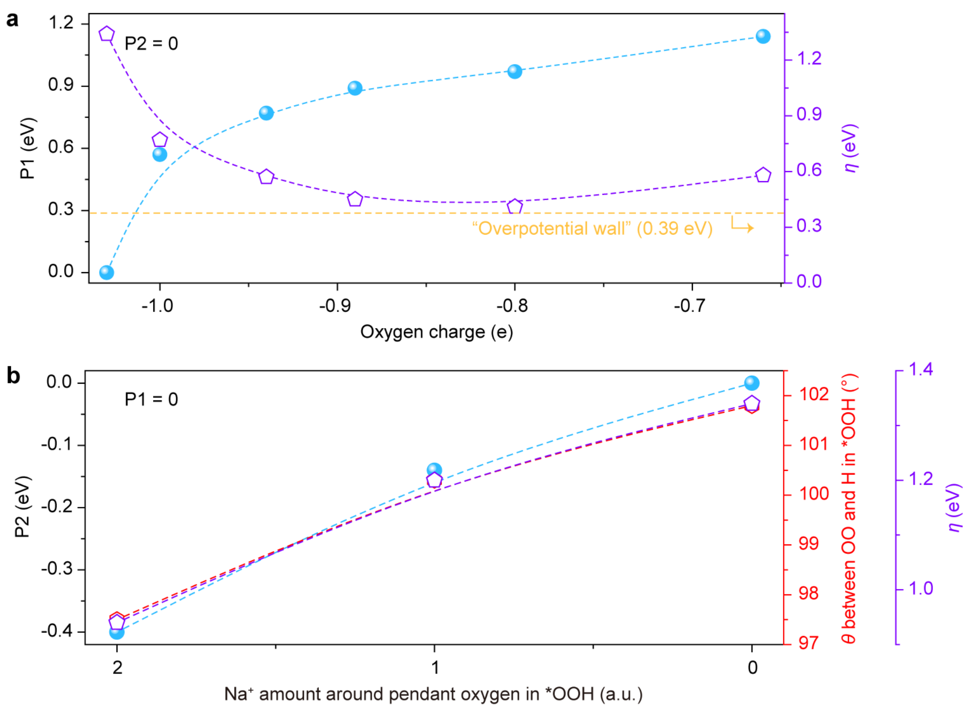

Supplementary Fig. 9 | a, Shifts in P1 to regulate the theoretical overpotential (η) by tuning oxygen charge. **b,** Shifts in P2 and θ to regulate the theoretical overpotential (η) by tuning Na^+-specific non-covalent interaction to circumvent LSR between *OOH and *OH.

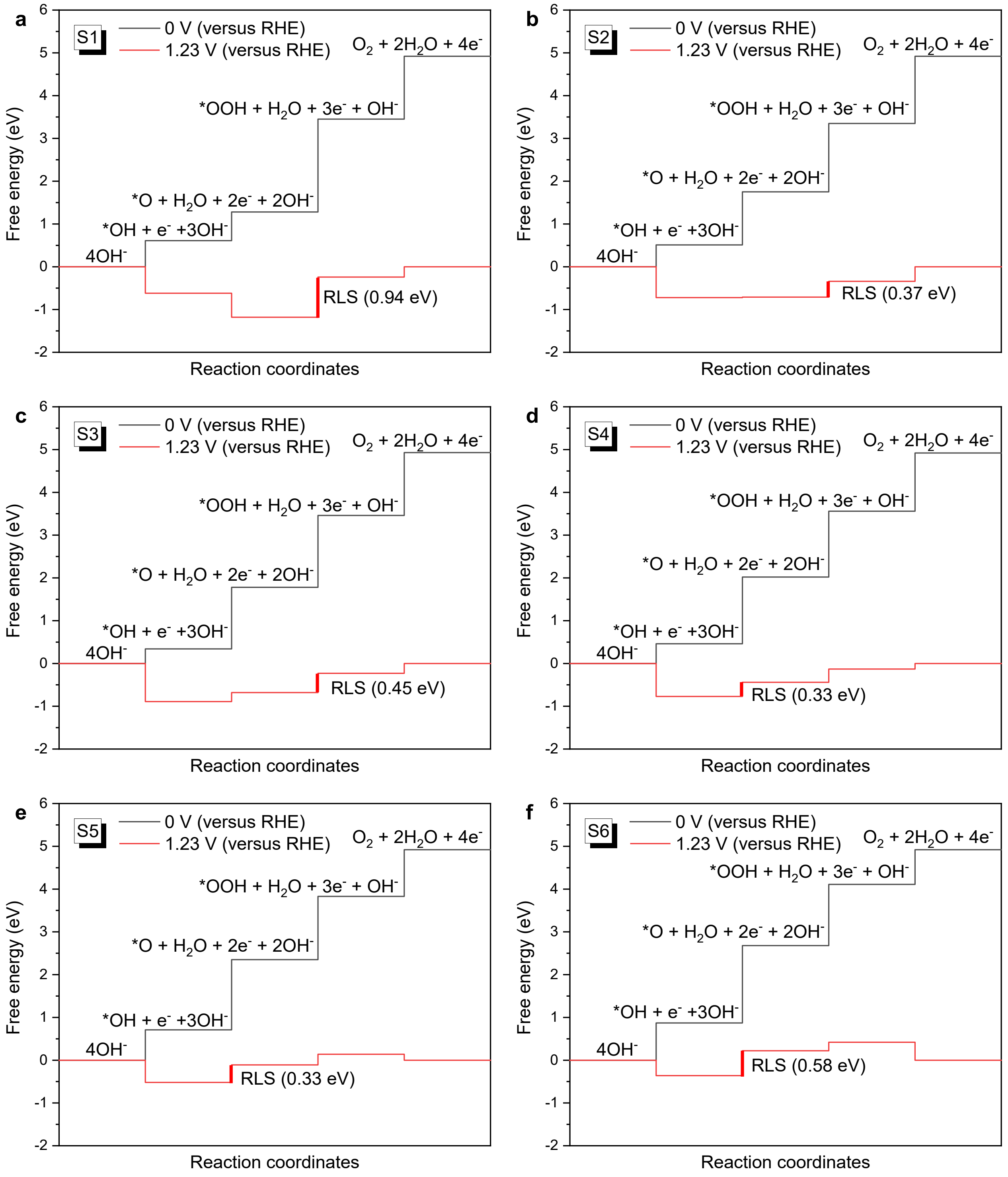

Supplementary Fig. 10 | Free energies of OER steps on different oxygen coordination environments in $Na_xMn_3O_7$ slabs under different potentials. (RLS represents rate-limiting step.)

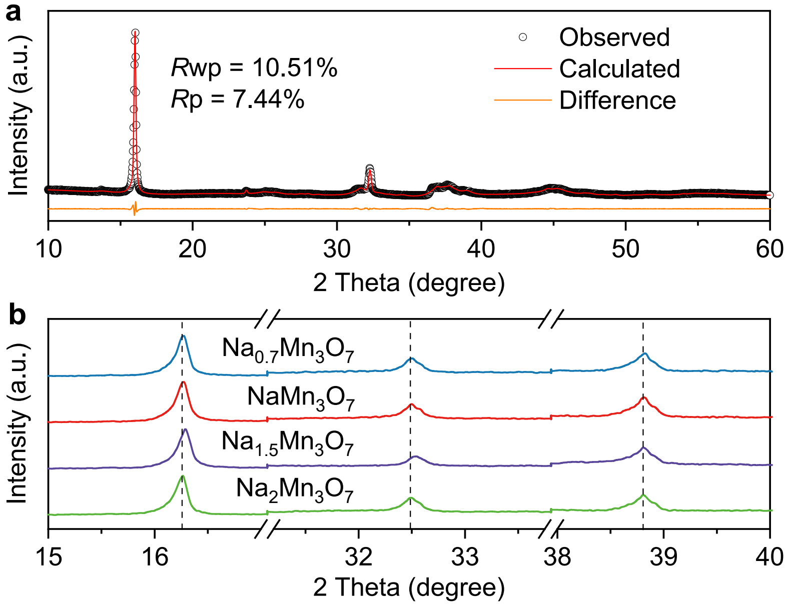

Supplementary Fig. 11 | **a,** Rietveld refinement of XRD pattern for $Na_2Mn_3O_7$. **b,** XRD patterns of as-synthesized $Na_xMn_3O_7$ materials.

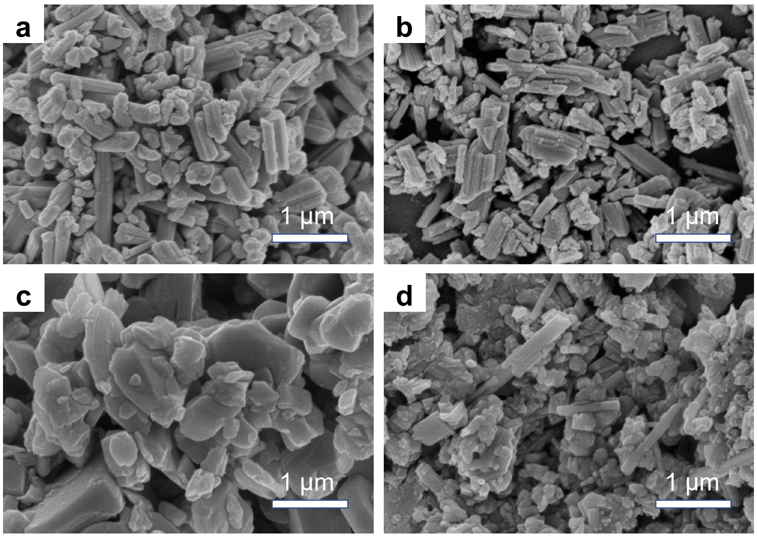

Supplementary Fig. 12 | SEM images of as-synthesized $Na_xMn_3O_7$ materials (**a,** $Na_2Mn_3O_7$, **b,** $Na_{1.5}Mn_3O_7$, **c,** $NaMn_3O_7$, **d,** $Na_{0.7}Mn_3O_7$).

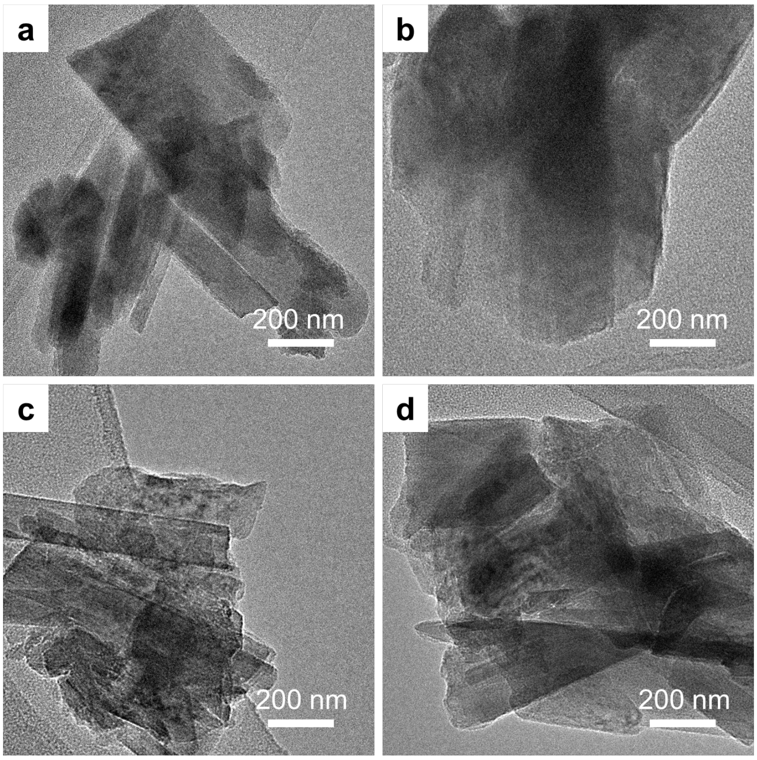

Supplementary Fig. 13 | HRTEM images of as-synthesized $Na_xMn_3O_7$ materials (**a,** $Na_2Mn_3O_7$, **b,** $Na_{1.5}Mn_3O_7$, **c,** $NaMn_3O_7$, **d,** $Na_{0.7}Mn_3O_7$).

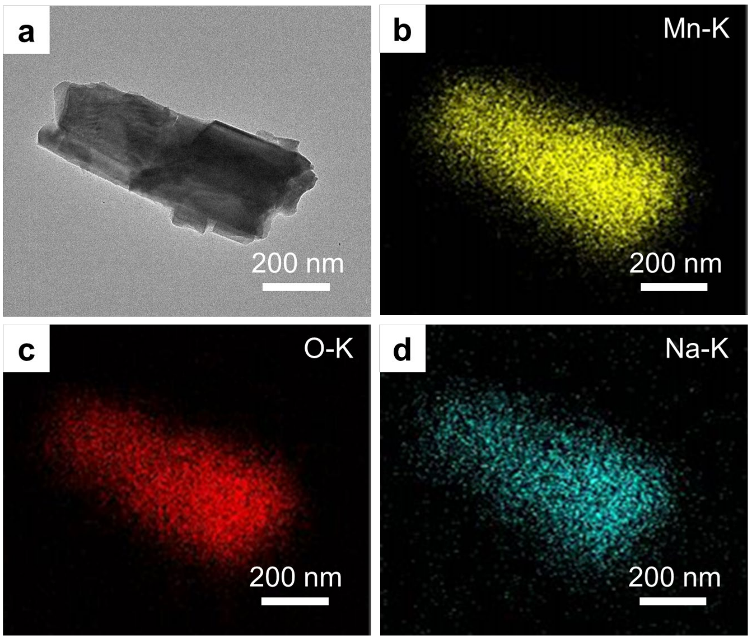

Supplementary Fig. 14 | TEM image and energy dispersive spectrometer (EDS) mapping images of $NaMn_3O_7$.

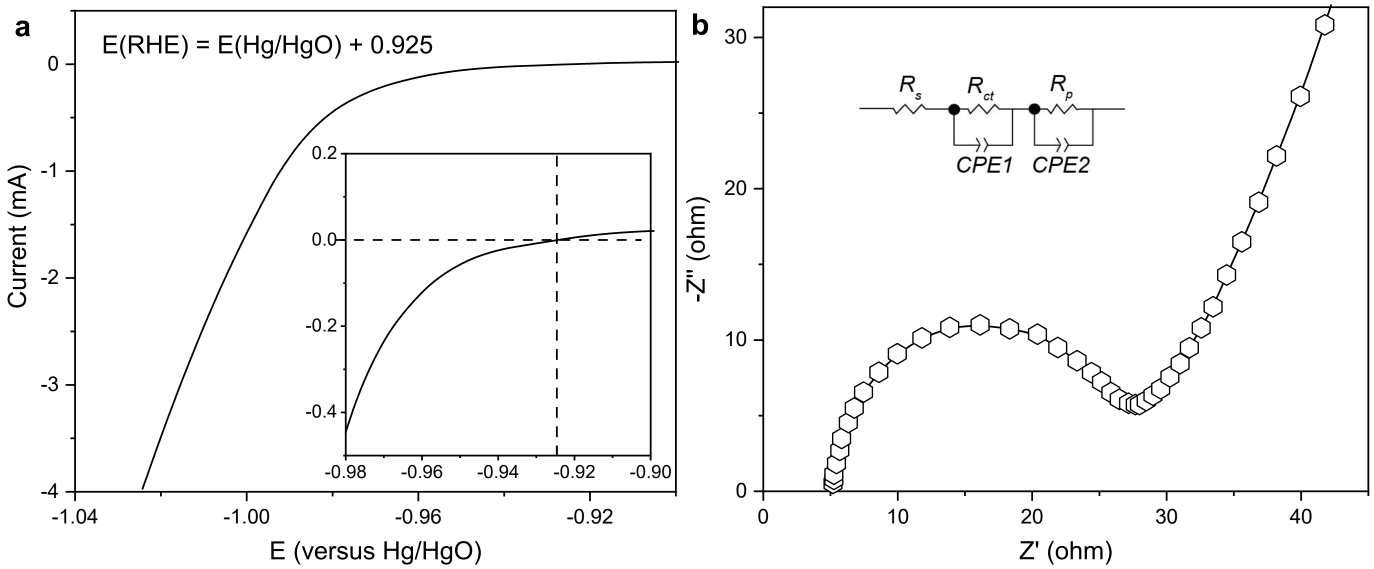

Supplementary Fig. 15 | a, Reversible hydrogen electrode (RHE) calibration. **b,** solution resistance measurement. Calibration of the Hg/HgO reference electrode was conducted in a standard three-electrode system at ambient temperature and in saturated H_2 atomosphere using 40 wt% Pt/C (0.250 mg cm^{-2}) and Pt foil as the working and counter electrode at a rotational speed of 1600 rpm[6].

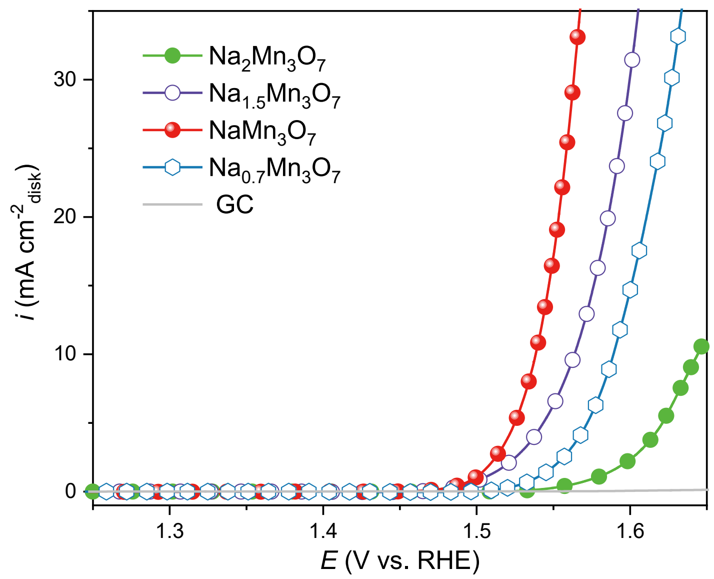

Supplementary Fig. 16 | Polarization curves (current normalized by geometric area of the disk) of the as-synthesized $Na_xMn_3O_7$. (The 2nd LSV curve is used for comparison.)

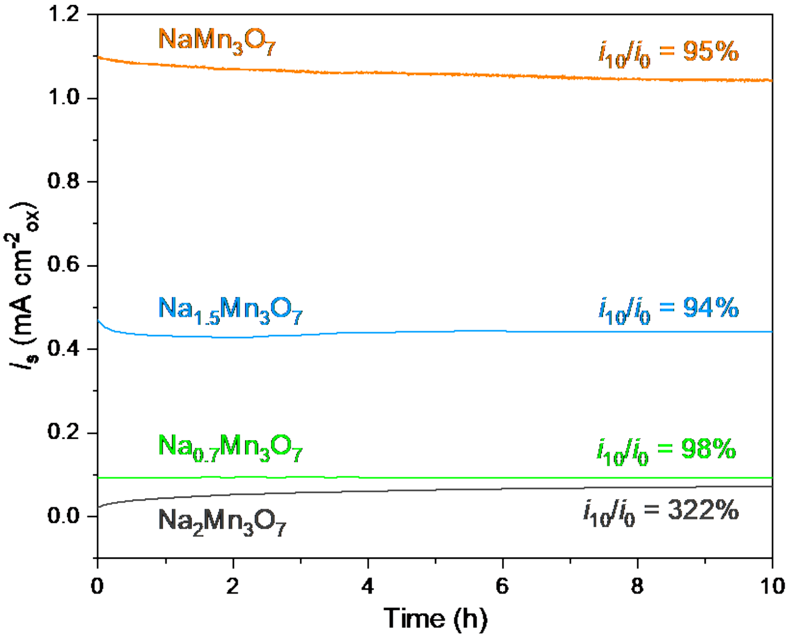

Supplementary Fig. 17 | Chronoamperometric curve of $Na_xMn_3O_7$ at 1.55 V versus RHE.

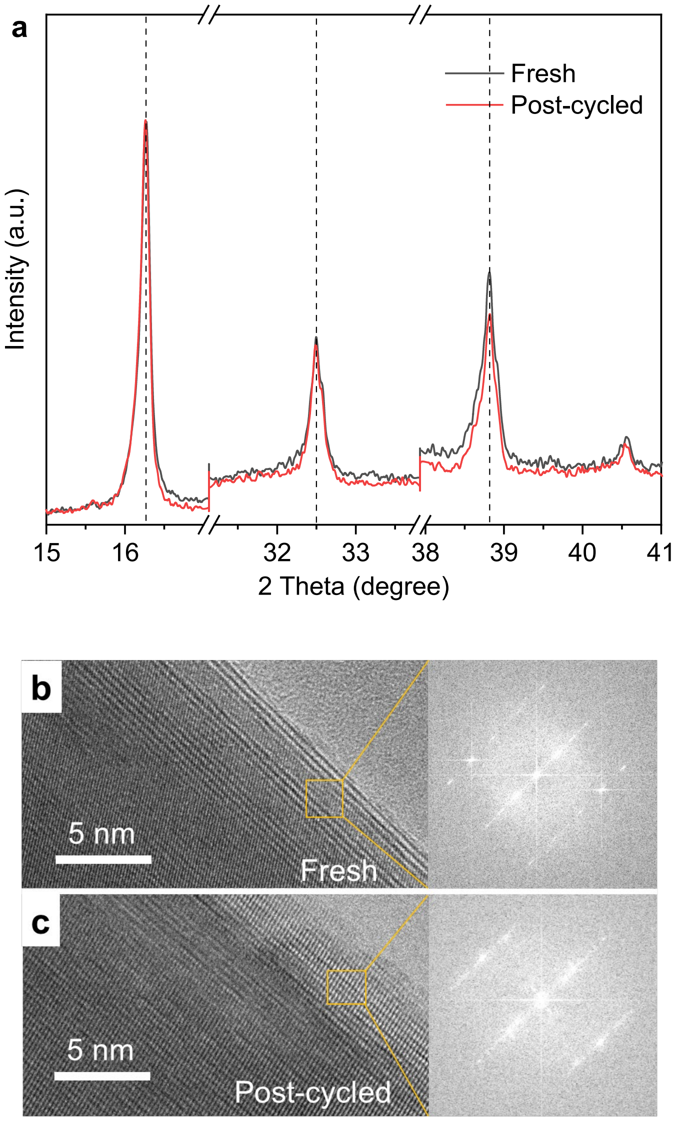

Supplementary Fig. 18 | a, XRD patterns of $NaMn_3O_7$ before and after OER measurement. **b, c,** HRTEM images and corresponding FFT patterns of $NaMn_3O_7$ before and after OER measurement.

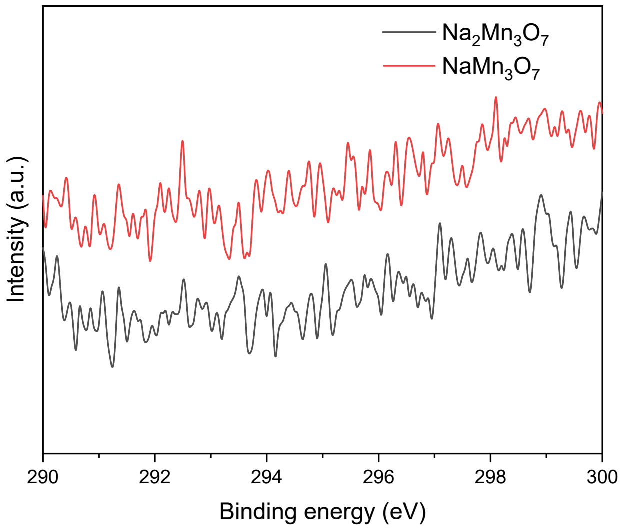

Supplementary Fig. 19 | XPS spectra of K 2*p* of $Na_2Mn_3O_7$ and $NaMn_3O_7$ after OER measurement.

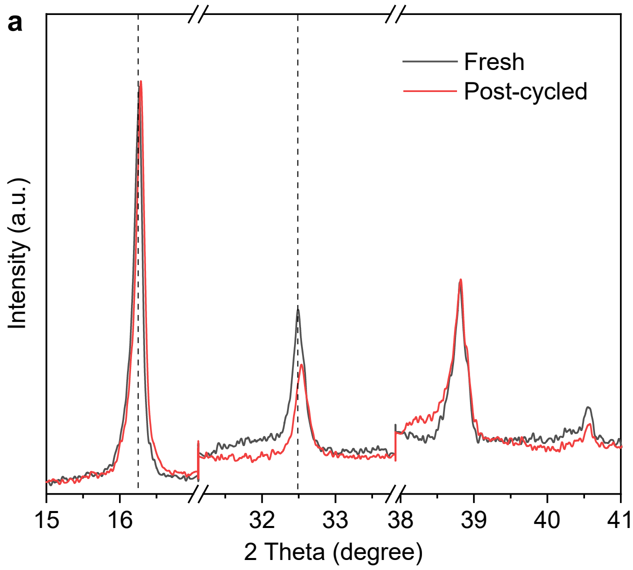

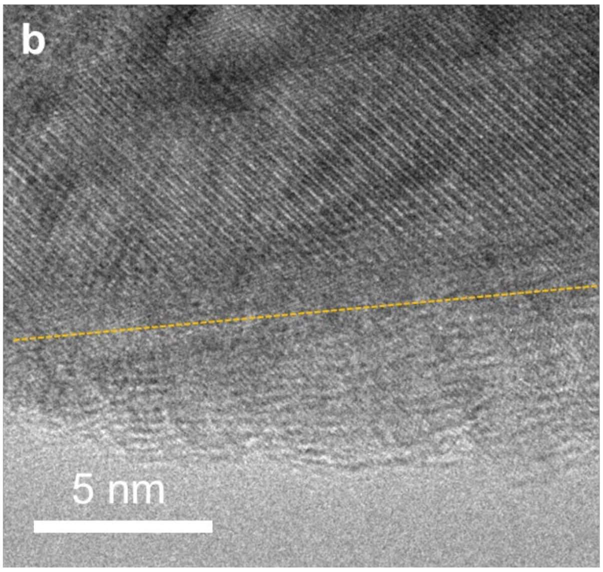

Supplementary Fig. 20 | a, XRD patterns of $Na_2Mn_3O_7$ before and after OER measurement. **b,** HRTEM of $Na_2Mn_3O_7$ after OER measurement.

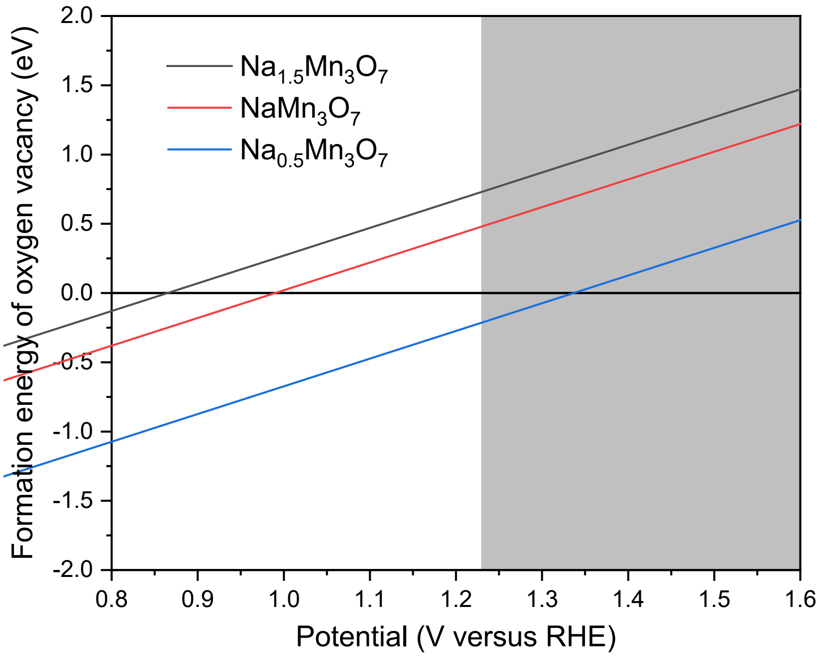

Supplementary Fig. 21 | Formation energy of oxygen vacancy in $Na_xMn_3O_7$ (x = 1.5, 1 and 0.5).

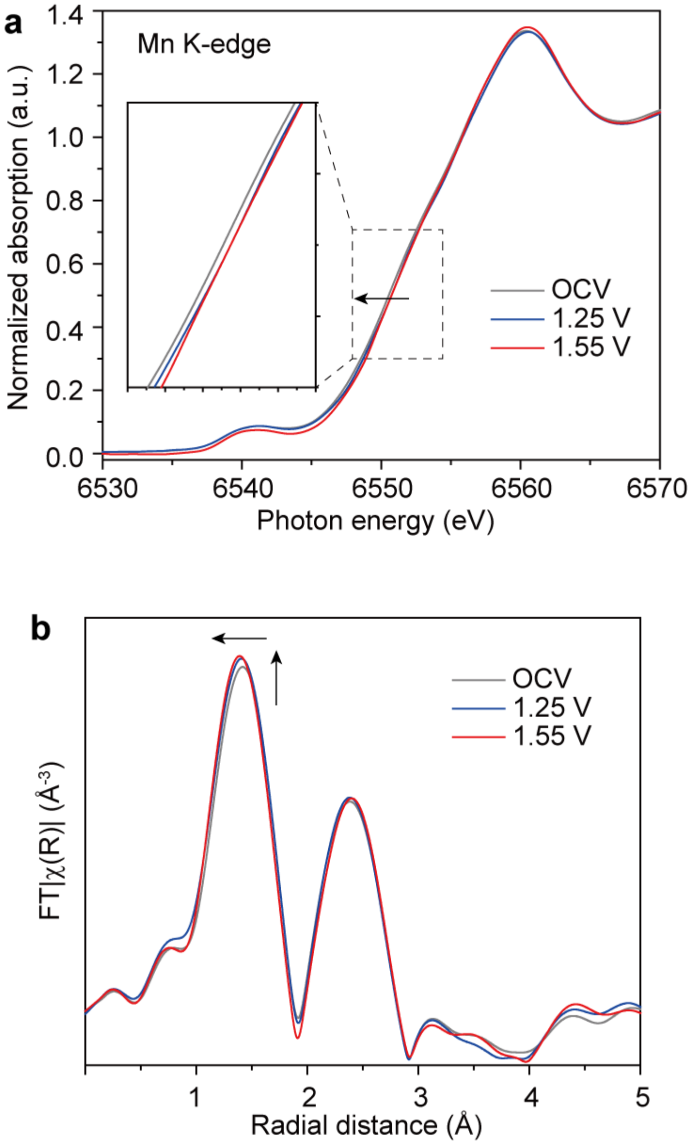

Supplementary Fig. 22 | Normalized *in situ* Mn K-edge XANES spectra (**a**) and Fourier transformed EXAFS $k^3\chi(k)$ oscillation functions (**b**) of $NaMn_3O_7$ under open circuit, 1.25 and 1.55 V (versus RHE).

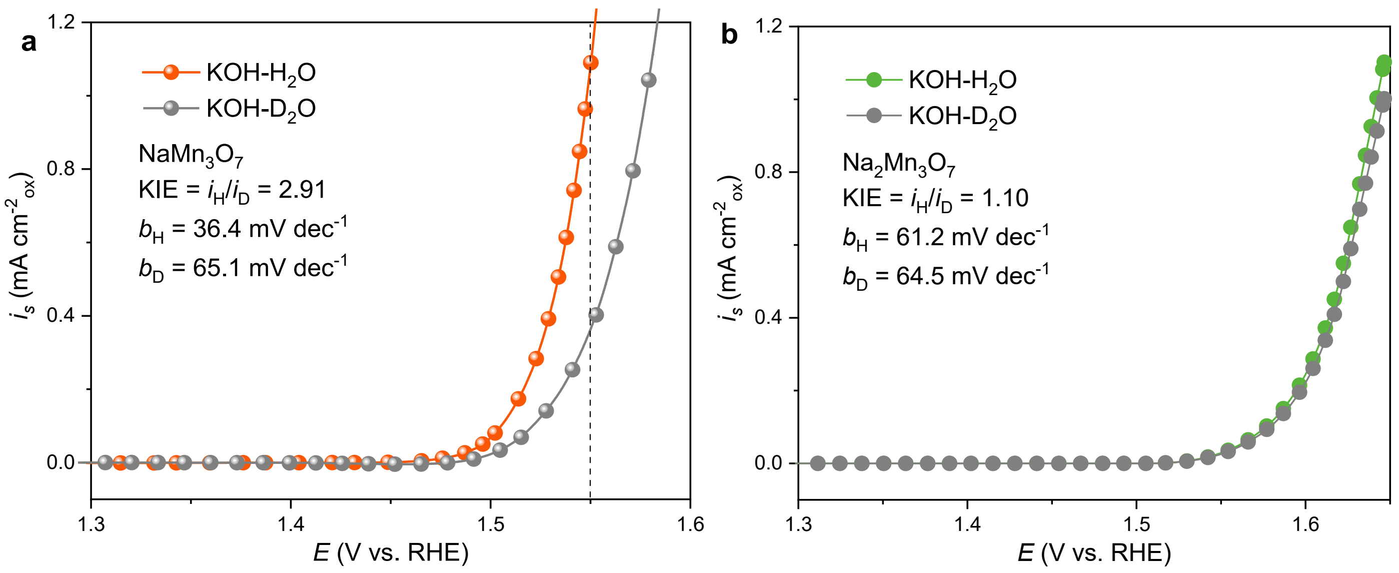

Supplementary Fig. 23 | Comparison of H/D kinetic isotope effect (KIE) on $NaMn_3O_7$ (**a**) and $Na_2Mn_3O_7$ (**b**) measured in H_2O and D_2O, respectively (the 2nd LSV curve is used for comparison.).

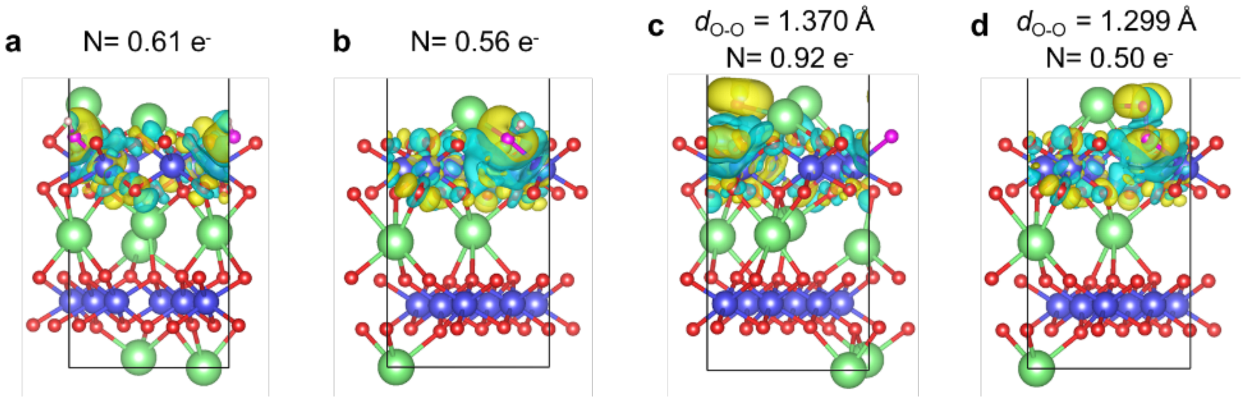

Supplementary Fig. 24 | Comparison of charge density difference of different adsorbed intermediates of *OH and *OO on $Na_2Mn_3O_7$ and $NaMn_3O_7$. Yellow and blue colours represent charge accumulation and depletion, respectively, with an iso-surface value of 0.0015 e$Å^{-3}$. N represents the number of transfered electrons between the adsorbed intermediate and catalyst surface.

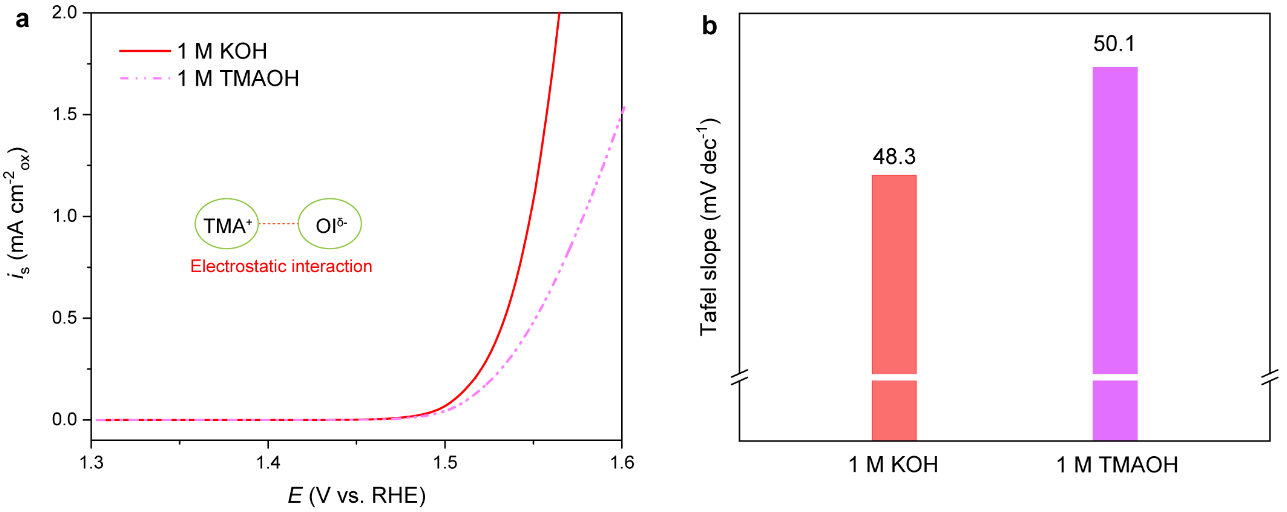

Supplementary Fig. 25 | Comparison of polarization curves (**a**) and derived Tafel slopes (**b**) of $NaMn_3O_7$ in 1 M KOH and 1 M TMAOH ($OI^{\delta-}$ represents as the negative oxygenated intermediates.).

Supplementary References

1 Zhao, Z.-J. *et al.* Theory-guided design of catalytic materials using scaling relationships and reactivity descriptors. *Nat. Rev. Mater.* **4**, 792-804 (2019).

2 Man, I. C. *et al.* Universality in oxygen evolution electrocatalysis on oxide surfaces. *ChemCatChem* **3**, 1159-1165 (2011).

3 Rong, X., Parolin, J. & Kolpak, A. M. A fundamental relationship between reaction mechanism and stability in metal oxide catalysts for oxygen evolution. *ACS Catal.* **6**, 1153-1158 (2016).

4 Nørskov, J. K. *et al.* Origin of the overpotential for oxygen reduction at a fuel-cell cathode. *J. Phys. Chem. B* **108**, 17886-17892 (2004).

5 Doyle, A. D., Montoya, J. H. & Vojvodic, A. Improving oxygen electrochemistry through nanoscopic confinement. *ChemCatChem* **7**, 738-742 (2015).

6 Wei, C. *et al.* Recommended practices and benchmark activity for hydrogen and oxygen electrocatalysis in water splitting and fuel cells. *Adv. Mater.*, e1806296 (2019).
